# Chromosome Number, Ploidy Level, and Nuclear DNA Content in 23 Species of *Echeveria* (Crassulaceae)

**DOI:** 10.3390/genes12121950

**Published:** 2021-12-03

**Authors:** Guadalupe Palomino, Javier Martínez-Ramón, Verónica Cepeda-Cornejo, Miriam Ladd-Otero, Patricia Romero, Jerónimo Reyes-Santiago

**Affiliations:** 1 Laboratorio de Citogenética, Jardín Botánico, Instituto de Biología, Universidad Nacional Autónoma de México, Mexico City 04510, Mexico; mramon@ib.unam.mx (J.M.-R.); miladdo53@gmail.com (M.L.-O.); 2 Laboratorio de Biotecnología Molecular y de Cultivos, EMA6, Facultad de Ciencias Biológicas, Benemérita Universidad Autónoma de Puebla, Puebla 72570, Mexico; veronica.cepeda@correo.buap.mx; 3 Instituto de Investigaciones en Matemáticas Aplicadas y en Sistemas, Universidad Nacional Autónoma de México, Mexico City 04510, Mexico; patricia@sigma.iimas.unam.mx; 4 Laboratorio de Sistemática Filogenética y Taxonomía Integrativa, Jardín Botánico, Instituto de Biología, Universidad Nacional Autónoma de México, Mexico City 04510, Mexico; jreyes@ib.unam.mx

**Keywords:** *Echeveria*, endemism, endopolyploid nuclei, flow cytometry

## Abstract

*Echeveria* is a polyploid genus with a wide diversity of species and morphologies. The number of species registered for *Echeveria* is approximately 170; many of them are native to Mexico. This genus is of special interest in cytogenetic research because it has a variety of chromosome numbers and ploidy levels. Additionally, there are no studies concerning nuclear DNA content and the extent of endopolyploidy. This work aims to investigate the cytogenetic characteristics of 23 species of *Echeveria* collected in 9 states of Mexico, analyzing 2*n* chromosome numbers, ploidy level, nuclear DNA content, and endopolyploidy levels. Chromosome numbers were obtained from root tips. DNA content was obtained from the leaf parenchyma, which was processed according to the two-step protocol with Otto solutions and propidium iodide as fluorochrome, and then analyzed by flow cytometry. From the 23 species of *Echeveria* analyzed, 16 species lacked previous reports of 2*n* chromosome numbers. The 2*n* chromosome numbers found and analyzed in this research for *Echeveria* species ranged from 24 to 270. The range of 2C nuclear DNA amounts ranged from 1.26 pg in *E. catorce* to 7.70 pg in *E. roseiflora*, while the 1C values were 616 Mbp and 753 Mbp, respectively, for the same species. However, differences in the level of endopolyploidy nuclei were found, corresponding to 4 endocycles (8C, 16C, 32C and 64C) in *E. olivacea*, *E. catorce*, *E. juarezensis* and *E. perezcalixii*. In contrast, *E. longiflora* presented 3 endocycles (8C, 16C and 32C) and *E. roseiflora* presented 2 endocycles (8C and 16C). It has been suggested that polyploidization and diploidization processes, together with the presence of endopolyploidy, allowed *Echeveria* species to adapt and colonize new adverse environments.

## 1. Introduction

*Echeveria* is a genus in the Crassulaceae family consisting of perennial plants that grow naturally in America [1]. The distribution of the *Echeveria* genus spans from west Texas to Argentina, across Mexico, Guatemala, and Central and South America [2,3,4,5,6]. The *Echeveria* genus includes a vast diversity of species which are commonly found on rocky cliffs in different ecosystems, such as pine and oak forests, cloud forests, and some xerophytic scrublands, which are mainly limited to temperate zones of Mexico [2,3,5]. Diversification of *Echeveria* species has been influenced by the isolation of the populations by geographical barriers caused for the rugged orography of Mexico [3]. Hidalgo, Mexico, Oaxaca, and Puebla are the states with the highest richness and endemism of this genus in Mexico [3,7]. The number of species registered for *Echeveria* genus is approximately 170, of which 143 (85%) are endemic to Mexico [3,8]. For this reason, Mexico is considered a diversification center for the genus *Echeveria*. 

The *Echeveria* genus includes perennials and succulent plants with a pentametric corolla of great beauty, short or elongated stems, leaves disposed spirally, without obvious or conspicuous petioles, with fibrous roots [1,3]. *Echeveria* species have great potential as ornamental plants and are appreciated in horticultural practices and as medicinal plants [2,9]. The genus *Echeveria* has been divided into 17 series, which were established based on morphological and chromosomal evidence, and have remained unchanged for almost 50 years [6,10].

In the Crassulaceae family, the chromosomes are very small, and it seems clear that many genera in this family originated from polyploids [1,5,11,12,13,14,15,16,17,18,19,20]. Cytological studies in more than 500 taxa of Crassulaceae have revealed high chromosome numbers and karyological variability, especially in the *Sedum* genus, from which the Mexican species *S. suaveolens* exhibits the highest chromosome number in Angiosperms with up to 2*n* = 640 [21]. The number of chromosomes in the genus *Echeveria* presents great diversity and more than 50 gametic numbers have been recorded by Uhl, from *n* = 12 − 34 to *n* = 260 in polyploid plants from different localities [5]. Due to the wide range in chromosome numbers (*n* and 2*n*), some of the species that have been described as diploids show a chromosome number as high as would be expected in polyploid plants [5,12,15,16,17].

Polyploidy accompanied speciation is in several families of angiosperms whose species have three or more basic chromosome sets (x) representing 1Cx holoploid genomes in their somatic cell nuclei [22,23]. *Opuntia* species (Cactaceae), where interspecific hybridization often occurs, are frequently polyploid plants [24,25], such as *Sedum* [26] and *Echeveria* [13,14,16,17], both of Crassulaceae family. Polyploidy is therefore considered one of the main causes of speciation and diversification in angiosperms [23,27,28,29,30,31,32].

The most common applications of flow cytometry in plants are the determination of genome size, polyploidy, and endopolyploidy [33,34,35,36,37,38]. The C-value for approximately 10,770 angiosperms was estimated and is frequently used in systematics and plant improvement studies [22,33,35,37,38,39,40]. It is reported as an interspecific variation of 2378 times the C-value of DNA in plants when comparing the lowest and the highest values [39]. 

The smallest 1C-values are observed in three species of the genus *Genlisea aurea* 0.065 pg (63.6 Mpb) [41] and *Genlisea tuberosa,* with 0.066 pg [42]. The largest 1C-value (152.23 pg) has been registered for *Paris japonica* (Melanthiaceae). The distribution of the genome size in several groups of plants is correlated with cytological, physiologic, and ecologic characters [43]. Recently, the data on C-values was used in studies about phylogenetic relations, and for the delimitation of taxa with the same chromosome number [44,45]. Studies about the content of nuclear DNA in polyploids have contributed to the knowledge of the mechanisms by which they are formed [24,27,28,46,47,48].

Endopolyploidy is an important phenomenon in plants in which chromosomal DNA replicates in the absence of mitosis, giving rise to cells with different levels of endopolyploidy (8C, 16C…) [33,49,50]. Endopolyploidy nuclei arise from a modification of the cell cycle where DNA of the chromosomes is replicated during the synthesis phase (S) but without further mitosis, so that the sister chromatids do not separate [51]. Consequently, a cycle is established between the synthesis phase and the G phases, known as an endocycle. With each endocycle, the amount of DNA doubles and cells with different levels of endopolyploidy are formed, depending on the number of endocycles each has passed through [50]. Plants showing endoreduplication are named polysomatic and have cells at different levels of polyploidy in their tissues and organs [33]. Such cells are referred to as endopolyploidy cells.

A consequence of endopolyploidization is the formation of cells with very large nuclei [52]. Furthermore, it is also suggested that stress caused by environmental changes, such as low or high light, temperature changes, water stress, etc., can induce endopolyploidy as part of a response that allows plants to adapt to these conditions and overcome the physical or genomic damage resulting from stress [50,53].

Endopolyploidy is more frequent in plants with small genomes, such as *Arabidopsis thaliana* [54] and annual plants rather than perennial ones [33]. The occurrence and the degree of endopolyploidy is documented in several groups of plants, such as algae, bryophytes, ferns, gymnosperms, and angiosperms [50]. In species of Cactaceae, such as *Mammillaria san*-*angelensis* and several species of *Opuntia* and *Nopalea*, endopolyploidy has been found at different levels [24,34,55]. Nevertheless, regarding the genus *Echeveria*, there are no publications on its DNA content, nor the existence of endopolyploidy; however, this information could provide useful knowledge for taxonomic studies and biotechnology, conservation, and floriculture programs for these species.

The goals of this study are: (1) to assess the interspecific variation in chromosome counts 2*n* and ploidy levels of the *Echeveria* species; (2) to evaluate the variation of the nuclear DNA content in the genus, since there are no previous studies regarding nuclear DNA content for any *Echeveria* species; (3) to evaluate the presence of endopolyploidy in the taxa included in the study.

## 2. Materials and Methods

### 2.1. Plant Material

In total, 25 wild populations of 23 *Echeveria* species were collected in 9 states of Mexico: Durango, Guerrero, Jalisco, Michoacan, Oaxaca, Puebla, San Luis Potosi, and Veracruz (Table 1). In the case of *Echeveria catorce* and *E. guerrerensis*, two distinct populations from San Luis Potosi and Guerrero, respectively, were analyzed (Table 1). Individuals from each species (5–10) were planted in pots containing organic soil, and a mix of inorganic substrate made of sand, tepojal (pumice stone), tezontle (red volcanic rock), and agrolite; plants were grown in greenhouse conditions at the Botanical Garden, Biology Institute, UNAM. Voucher specimens were deposited in the National Herbarium (MEXU) of the same Institution. Studied species correspond to 7 of the 17 series that comprise the *Echeveria* genus [6,7,56]. *Echeveria catorce* has not been formally described yet, however, it is widely recognized in the literature [19,56]. 

### 2.2. Mitotic Chromosome Counts 

Based on 5–10 individual plant samples of each population of *Echeveria*, 9 mitotic cells at the metaphase stage were observed for the determination of chromosome numbers (2*n*). Elongated secondary root tips were collected in the morning and placed in 8-hydroxyquinoline 0.002 M solution for 6 hours at 18 °C in the dark. After wards, the root tips were fixed in fresh Farmer´s solution (three-parts absolute ethanol: one-part glacial acetic acid) for 24 h at 18 °C. The root tips were hydrolyzed in hydrochloric acid 1 N for 11 min at 60 °C and transferred to Schiff reagent for 1 h, and then to 1.8% propionic orcein to stain the chromosomes for 25 min following the procedure of García [57]. An additional treatment was required in some species with very hardened tissue—10% pepsin (Sigma) at 37 °C for 90 minutes; this treatment was used after staining with Schiff´s reagent. Slides were prepared and frozen with dry ice [58], dehydrated in absolute alcohol, and mounted in Canada balsam. Nine of the best cells from each plant were photographed using an Axio Vision Rel. 4.7 camera in a Zeiss photomicroscope III (Gottingen, Germany).

Basic chromosome number (*x*) was assigned based on previous literature evidence for related species [11,12,13,14,15,16,17,18,19,20].

### 2.3. Estimation of Nuclear DNA Content

Of the 23 studied *Echeveria* species. three to five young plants collected from the wild and kept in a greenhouse while they were analyzed and used to estimate nuclear DNA content, utilizing flow cytometry. Three replicates of each individual plant were analyzed. Internal standards for the genome size estimates consisted of *Solanum lycopersicum* cv. Stupické polni rané, 2C DNA = 1.96 pg [37]; *Zea mays* cv. CE-777, 2C DNA = 5.43 pg and *Pisum sativum* L. cv. Ctirad, 2C DNA = 9.09 pg [37]. Samples were prepared according to the two-step protocol with Otto solutions as detailed in Dolezel et al. [37], with some modifications.

Leaf tissue from *Echeveria* plants was used for the analysis of DNA content, from which the waxy cuticle had been previously removed to prevent the nuclei from adhering to each other or to the surface of the containers (due to their wax content). The waxy cuticle was removed using a razor blade; a shallow cut was made at the edge of the leaf and then the cuticle was pulled and separated with the help of the same blade and fine-tipped pins. Due to the difficulty of obtaining isolated nuclei of *Echeveria* and the great differences in the content of nuclei between the *Echeveria* leaves and the internal standards, it was almost impossible to obtain a good relative proportion of nuclei. Therefore, it was decided to use the pseudo-standardization technique, where the nuclei solutions are prepared separately, check Temsch et al. [59]. Between 120 and 250 mg of *Echeveria* leaf tissue was chopped with a razor blade in a Petri dish containing 1.5 to 2.00 mL of Otto 1 solution (1.5 mL of 0.1 M citric acid and 0.5% Tween 20), and then filtered through a 50 µm nylon mesh. Separately, 20–42 mg of internal standard plant was chopped into another Petri dish containing 1 mL of Otto1 solution and filtered in the same way [59]. Both solutions were incubated for 15 min at room temperature. Then, samples were pelleted by centrifugation (90 g for 3 min), and each was suspended in 500 µL of Otto 1 solution. 

Subsequently, 250 µL of each nuclei solution (*Echeveria* and Internal plant) was poured into a sample tube and 2 mL of 0.4 M Na_2_HPO_4_ (Otto 2 solution) was added to the suspension along with stock solutions of propidium iodide and RNase, both at 50 µg mL^−1^. 

The definitive sample was filtered through a 50 µm nylon mesh (to avoid the passage of possible crystals formed in the Otto 2 solution, given its high salt concentration, which could cause obstruction in the cytometer conduits) and then analyzed using a Partec CyFlow SL Cytometer (equipped with a 488 nm solid state laser). The instrument gain was adjusted so that the peak representing either the G_1_ nuclei of *Echeveria* or the G_1_ nuclei of the internal standard was placed in channel 50 of a 250-channel linear scale.

At least 10,000 nuclei were analyzed for each sample. Peak means areas and coefficient of variation (CV%) were obtained for each peak of interest (sample and standard) using the gating function in the FloMax software for cytometry (Partec). The CV accepted for the samples was less than 5.00%; for the case of the internal standards, values between 2.80% and 3.72% were obtained, while for *Echeveria* the range was between 3.83% and 4.98%. Nuclear genome size was calculated according to Dolezel et al. [37] using the formula:

A = (B/C) × D(1)

where A = *Echeveria* 2C nuclear DNA content (pg); B = *Echeveria* G_0_/G_1_ peak mean; C = internal standard G_0_/G_1_ peak mean; and D = 2C DNA content internal standard.

The 1Cx-value was calculated for all the studied species by dividing nuclear DNA content by the ploidy level, as suggested by Greilhuber et al. [39] and multiplying it later by 978 to convert it to Mbp [37].

### 2.4. Endopolyploidy Determination

Leaf tissue without the waxy cuticle was also used for endopolyploidy determination and the same procedure was carried out for the determination of the nuclear DNA content, but without the addition of an internal standard. The gain of the Partec CyFlow SL Cytometer was adjusted so that all the peaks could be seen on a logarithmic scale. The nuclei number and coefficient of variation (CV) were obtained for each peak using the gating function in the FloMax Software by Partec.

The cycle value or endoreduplication index was calculated according to procedure described by Barow, 2003 [60], using the formula:

Cycle value = (0 × n2C + 1 × n4C + 2 × n8C+⋯)/(n2C + n4C + n8C+⋯)(2)

where, n2C + n4C + n8C, corresponded to the nuclei number of each level C, 2C, 4C, 8C, 16C, 32C, and 64C. Plants with tissues showing values greater than 0.1 of the Cycle values are considered polysomatic [60,61].

### 2.5. Statistical Analyses

A one-way variance analysis (ANOVA) was conducted to compare the chromosome numbers of the series Gibbiflorae, Angulatae, and Racemosae. Data were transformed to reach normality and variance homogeneity by applying a Box–Cox transformation calculated by the JMP program. The algorithm calculated to produce the best transformation was: *x* = [(2N^−0.4^) × (−1)]/(−0.0013246), where *x* = data with transformation, and N = data without transformation.

Also, a Tuckey–Kramer test comparison was performed among groups in this series. However, the series Echeveria, Nudae, Pruinosae, and Urbinae contains only one species and it was not possible include in the ANOVA analyses.

Differences between the DNA content values (pg) of *Echeveria* populations were evaluated through a bi-factorial nested variance analysis (Nested ANOVA model). Random effects of repeated measurements on the same individual were considered. Natural logarithm transformed values of the response variable in the ANOVA model was significant, as well as each of its components. Multiple mean comparison test analyses of the DNA 2C content result values were done to compare the 25 populations of the 23 species of *Echeveria* based on a Tukey–Kramer test, a post-hoc test conducted after ANOVA. Mean and standard errors are reported in the original scale.

Pearson correlation and regression analyses were applied to evaluate the correlation among polyploidy levels and the 2*n* number, and between the ploidy level and the 1C*x*-value. 

The comparison of the cycle value between the study species was carried out using a one-way variance analysis (ANOVA). The normality of the studentized residuals was verified and the homogeneity of the variances was verified. A post-hoc Tuckey–Kramer test was applied after the one-way ANOVA.

All statistical analyses were done with JMP 8.0 and JMP 10 (SAS Institute, Cary, NC, USA) and using the software R (R: A language and environment for statistics computing. R Foundation for Statistical Computing, Vienna, Austria, URI, https://www.R-project.org (accessed on 17 July 2021) [62].

## 3. Results

### 3.1. Endemism, Chromosome Numbers, and Ploidy Level

Chromosome numbers of 23 *Echeveria* species were analyzed in this research. Of them, sixteen were endemic, corresponding to 69.6% of the total species analyzed here (Table 2). The largest diversity and endemism of this genus were observed in Oaxaca. 

**Figure 1:**
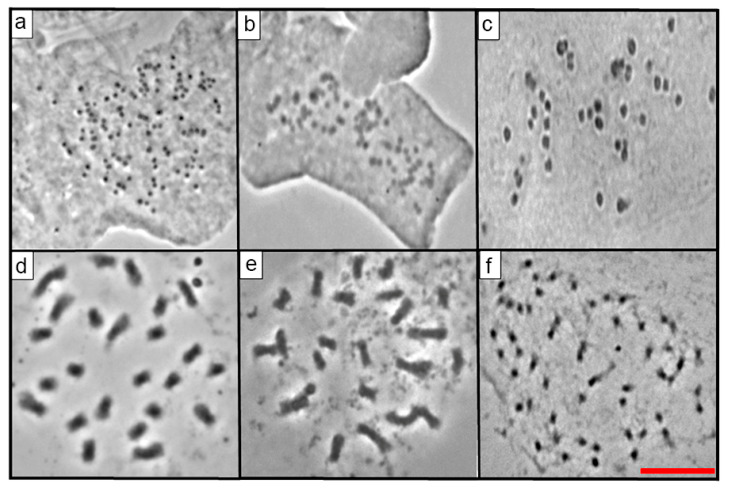
(**a**) *Echeveria altamirae* 2*n* = 108, (**b**) *E. caamanoi* 2*n* = 60, (**c**) *E. carnicolor* 2*n* = 36, (**d**) *E. catorce* 2*n* = 24 (Accession number JE-5469), (**e**) *E. catorce* 2*n* = 24 (Accession number EK-3223), (**f**) *E. cuicatecana* 2*n* = 60. Scale equals 10 μm.

**Figure 2:**
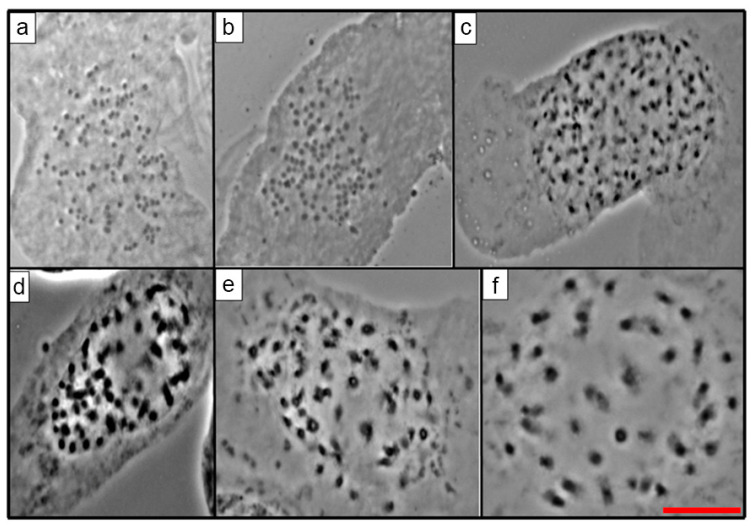
(**a**) *E. cupreata* 2*n* = 108, (**b**) *E. dactylifera* 2*n* = 108, (**c**) *E. gibbiflora* 2*n* = 172, (**d**) *E. guerrerensis* 2*n* = 54 (Accession number JE-7521), (**e**) *E*. *guerrerensis* 2*n* = 54 (Accession number JE-7526), (**f**) *E. helmutiana* 2*n* = 42. Scale equals 10 μm.

**Figure 3:**
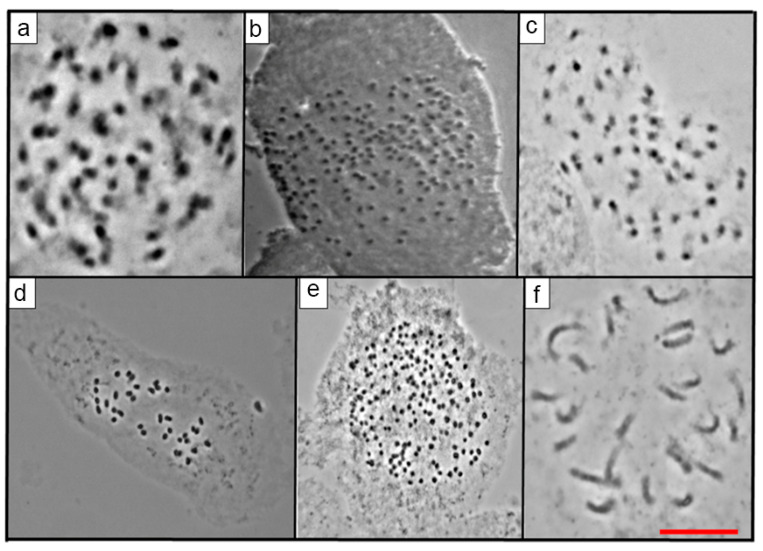
(**a**) *E. juarezensis* 2*n* = 54, (**b**) *E. longiflora* 2*n* = 162, (**c**) *E. magnifica* 2*n* = 54, (**d**) *E. multicaulis* 2*n* = 32, (**e**) *E. novogaliciana* 2*n* = 172, (**f**) *E. olivacea* 2*n* = 28. Scale equals 10 μm.

**Figure 4:**
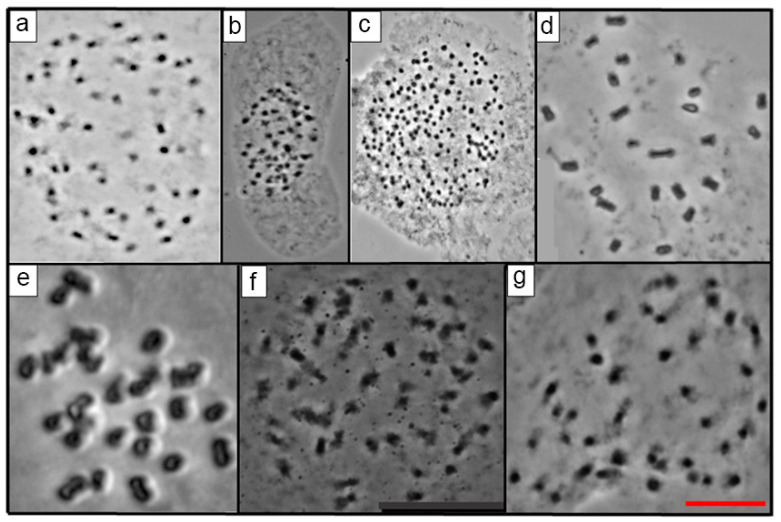
(**a**) *E. pallida* 2*n* = 54, (**b**) *E. perezcalixii* 2*n* = 54, (**c**) *E. roseiflora* 2*n* = 270, (**d**) *E. schaffneri* 2n = 24, (**e**) *E. triquiana* 2*n* = 32, (**f**) *E. Uhlii* 2*n* = 54 and (**g**) *E. zorzaniana* 2*n* = 40. Scale equals 10 μm.

There were different values of 2*n* and *x* in the 23 species of *Echeveria,* as expected in a genus known to be polybasic. For the first time in this research, the 2*n* for 16 species of *Echeveria* was reported (Table 2; Figure 1, Figure 2, Figure 3 and Figure 4), which ranged from 24 to 270. On the other hand, the most frequent *x* value was *x* = 27, present in 56.5% of the species was also the higher basic chromosome number (Table 2). The lowest value of x and 2*n* was observed in two diploid populations of *E. catorce* and of *E. schaffneri* with 2*n* = 2*x* = 24, and both species with *x* = 12. 

From the 23 species studied, 14 of them were diploid (60.86%), three were tetraploids (13.04%), two were pentaploids (8.70%), and one was a hexaploid (4.35%) (Table 2, Figure 1, Figure 2, Figure 3 and Figure 4). *E. roseiflora* was decaploid, which corresponds to 4.35% of the total number of species studied (Table 2, Figure 4c). Two species were polyploid–aneuploid and they represent the 8.70% of the total of species studied (Table 2).

With reference to the different series, from the 23 species of *Echeveria* studied here, 13 belong to the series Gibbiflorae. Six were diploids: *E. guerrerensis*, *E. juarezensis*, *E. magnifica*, *E. pallida*, and *E. perezcalixii*, all of them with 2*n =* 2*x* = 54. *E. triquiana* had 2*n* = 2*x* = 32. Three species were tetraploid, including *E. altamirae*, *E. cupreata.* and *E. dactylifera*, where 2*n* = 4*x* = 108. Meanwhile, *E. longiflora* was a hexaploid with 2*n* = 6*x* = 162 and *E. roseiflora* was a *decaploid* with 2*n* = 10*x* = 270. Two species were polyploid–aneuploid: *E. gibbiflora* with 2*n* = 6*x* + 10 = 172 and *E. novogaliciana* with 2*n* = 6*x* + 14 = 176. The last three species corresponded to the species with the highest chromosome numbers in the series Gibbiflorae (Table 1 and Table 2; Figure 1, Figure 2, Figure 3 and Figure 4).

Four species of the 23 analyzed belong to the Racemosae series: *Echeveria carnicolor* (2*n* = 2*x* = 36), *E. helmutiana* (2*n* = 2*x* = 42), *E. olivacea* (2*n* = 2*x* = 28), and *E. uhlii* (2*n* = 2*x* = 54); the last value is equal to that observed in several species of the series Gibbiflorae (Table 1 and Table 2; Figure 1 and Figure 2). The series Angulatae was represented by two diploid species: *E. schaffneri* (2*n* = 2*x* = 24), and *E. catorce* (2*n* = 2*x* = 24). With two populations analyzed in this research, the chromosome number presented by the last species was the lowest value reported in this study (Table 1 and Table 2).

The series Pruinosa, Urbinae, Nudae, and Echeveria were represented by a single species in each case, which in that order correspond to *E. cuicatecana* (2*n* = 5*x* = 60) and to *E. caamanoi* (2*n* = 5*x* = 60), which were pentaploids, to *E multicaulis* (2*n* = 2*x* = 32) and *E. zorzaniana* (2*n* = 2*x* = 40), both diploids. (Table 2).

The differences among the series concerning chromosome numbers were significant among Gibbiflorae, Racemosae, and Angulatae (ANOVA: *F*_2,18_ = 20.28, *p* < 0.0001). The Tukey–Kramer test comparisons among the series showed that the mean of the chromosome numbers of the Gibbiflorae series ($\bar{x}$ = 104.3 ± 15.6) was significantly higher than the mean of chromosome numbers of the Racemosae series ($\bar{x}$ = 40 ± 29.2). Similarly, the mean of the chromosome numbers of the Racemosae series was significantly higher than Angulatae series ($\bar{x}$ = 24 ± 33.7) (Table 3).

### 3.2. Nuclear DNA Content 

**Figure 5:**
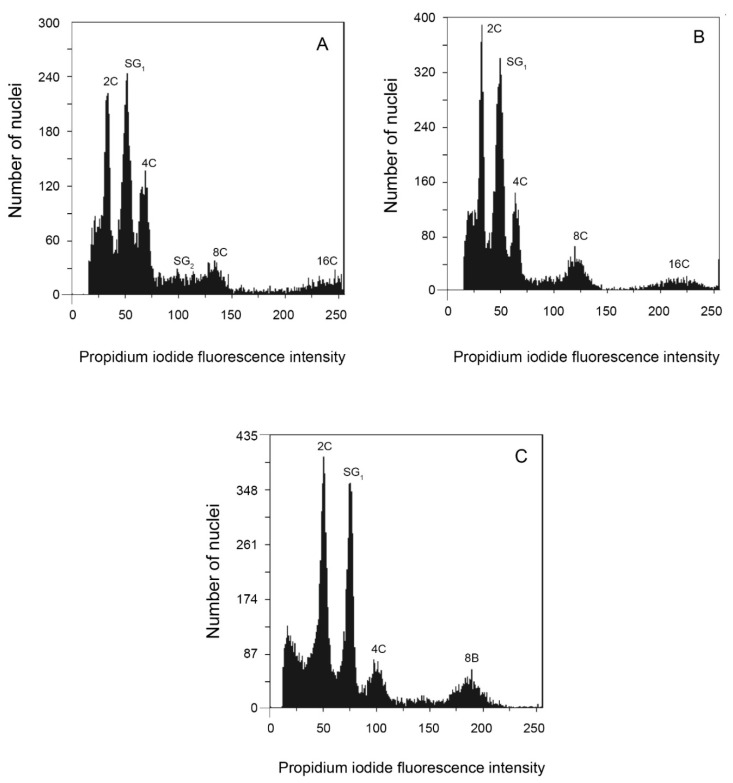
Nuclear DNA content estimation for 3 species of *Echeveria.* (**A**) Isolated nuclei analysis of *E. juarezensis* (2*n* = 2*x* = 54); peaks corresponding to nuclei 2C, 4C, 8C, and 16C from *E. juarezensis* are shown, peaks SG1 and SG2 represent nuclei from *Solanum lycopersicum*, used as the Internal standard. (**B**) Isolated nuclei analysis of *E. altamirae* (2*n* = 4*x* = 108); peaks corresponding to nuclei 2C, 4C, 8C and 16C from *E. altamirae* are shown; peak SG1 represents nuclei from *Zea mays*, used as the internal standard. (**C**) Isolated nuclei analysis of *E. novogaliciana* (2*n* = 6*x* = 176); corresponding to nuclei 2C, 4C, and 8C from *E. novogaliciana* are shown; peak SG1 represents nuclei from *Pisum sativum* used as the internal standard.

Regarding nuclear DNA amount, of the 13 species that belong to the Gibbiflorae series, 2C values were observed ranging from 1.31 pg in *E. juarezensis* to 7.70 pg in *E. roseiflora*. In the species of the Racemosae series, the 2C values were from 1.96 pg in *E. olivacea*, to 2.86 pg in *E. helmutiana*. In the Angulatae series, the two included species presented close values, with 1.26 pg and 1.29 pg in the 2 populations of *E. catorce* and 1.50 pg in *E. schaffneri*. On the other hand, *E. multicaulis* from the Nudae series presented a 2C value of 1.40 pg, very similar to *E. zorzaniana* with 1.49 pg that belongs to the series Echeveria. Finally, in *E. caamanoi* from the series Urbinae, the DNA content was 1.95 pg, and in *E. cuicatecana* from the series Pruinosae, it was 2.44; these values were close to some species from the Gibbiflorae series and the Racemosae series (Table 2, Figure 5).

Based on the 2C value (pg) of the 23 species of *Echeveria* analyzed by flow cytometry, one-way ANOVA revealed significant differences among species (α = 0.05, *p* < 0.0001). Ten significantly different groups were observed in the Tukey–Kramer test. The first group included *Echeveria roseiflora*, the second group was represented by *E. novogaliciana*, and the third group corresponded to *E. gibbiflora* and *E. altamirae.* Each group had statistically different means (Table 2). There were no significant differences among the next five groups (see central part in Table 2), which corresponded to *Echeveria perezcalixii*, *E. dactylifera*, *E. guerrerensis* (population JE-7521), *E. helmutiana*, *E. pallida*, *E. guerrerensis* (population JE-7526), *E. longiflora*, *E. cupreata*, *E. cuicatecana*, *E. uhlii*, *E. carnicolor* and *E. triquiana*. On the other hand, *Echeveria olivacea* and *E. caamanoi* composed the ninth group. The tenth group included six species: *Echeveria schaffneri*, *E. zorzaniana, E. multicaulis*, *E. magnifica*, *E. juarezensis,* and *E. catorce* (populations JE-5469 and EK-3223). The species in this last group presented the lowest DNA 2C-values of their genomes (Table 2).

### 3.3. Endopolyploidy 

**Figure 6:**
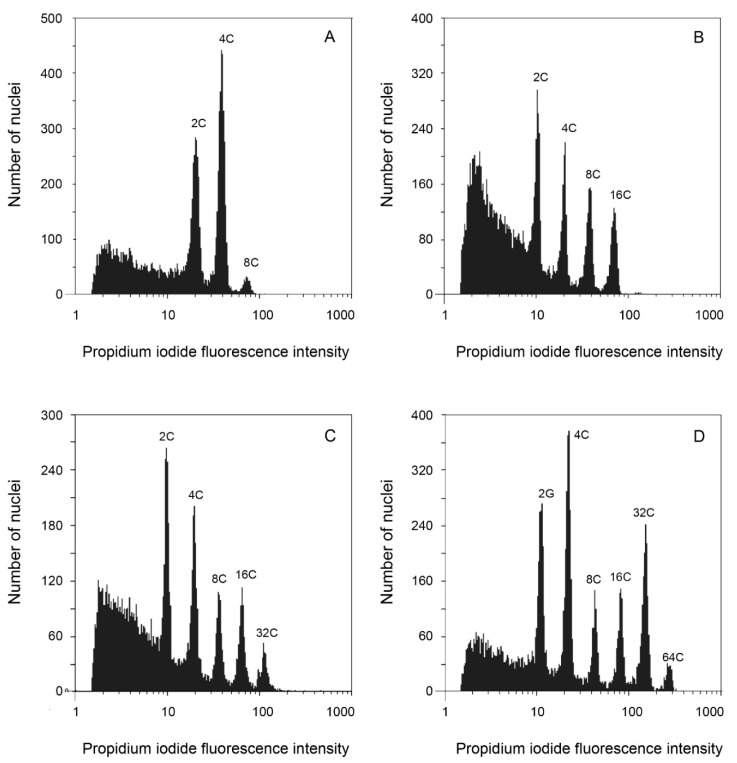
Endopolyploidy pattern in leaf parenchyma of four *Echeveria* species. *X*-axis is displayed in logarithmic scale. (**A**) *E. roseiflora* endopolyploidy pattern showing one endocycle (**B**) *E. altamirae* endopolyploidy pattern showing two endocycles. (**C**) *E. gibbiflora* endopolyploidy pattern showing three endocycles. (**D**) *E. caamanoi* endopolyploidy pattern showing four endocycles.

Differences in the percentage of endopolyploidy nuclei and number of endocycles were observed in the leaf parenchyma of 23 species of *Echeveria* analyzed (25 populations). From which, 4 species, *Echeveria novogaliciana*, *E. multicaulis*, *E. triquiana* and *E. altamirae,* presented 2 endocycles in all the analyzed individuals, which corresponds to 8C and 16C. Ten species presented 3 endocycles: 8C, 16C, and 32C, showing an endopolyploidy level of up to 32C DNA in all the individuals that were analyzed; these species correspond to *E. helmutiana*, *E. uhlii*, *E*. *magnifica*, *E. carnicolor*, *E. zorzaniana*, *E. gibbiflora*, *E. dactylifera*, *E. pallida*, *E. cupreata*, and one of the populations of *E*. *guerrerensis* (Accession number 7521). Five species: *E. schaffneri*, *E. cuicatecana*, *E.* *caamanoi*, *E. catorce* (Accession number 3223), and *E. guerrerensis* (Accession number 7526), presented 4 endocycles: 8C, 16C, 32C, and 64C, showing an endopolyploidy level of up to 64C in all the analyzed individuals (Table 4, Figure 6). 

Among the species analyzed, there were some that presented two endopolyploidy levels. The variation was detected between organisms of the same species and population. From these, *E. olivacea*, *E. juarezensis*, *E. perezcalixii*, and one of the populations of *E. catorce* (Accession number 5469), presented nuclei with 3 (8C, 16C, and 32C) or 4 (8C, 16C, 32C, and 64C) endocycles, while *E. longiflora* presented 2 (8C and 16C) or 3 (8C, 16C, and 32C) endocycles, and *E. roseiflora* showed 1 (8C) or 2 (8C and 16C) endocycles (Table 5). The distribution of the relative percentage of endopolyploid nuclei populations was variable, even among species with the same endopolyploidy level. (Table 4.). The variation in the number of endocycles and in the relative nuclei distribution in each C-level was reflected in the cycle value, which show values from 0.690 to 2.562, which is an adequate parameter to compare the degree of endopolyploidy [64] (Table 4 and Table 5).

The results of the one-way ANOVA analysis corresponding to the value of the cycle of the 23 species (25 populations), showed significant differences between the species (*F* _24,33_ = 6.8799, *p* < 0.0001). The post hoc Tuckey–Kramer test shows five groups. The first group included *E. schaffneri*, *E. catorce* (Accession number 3223), and *E. cuicatecana* ($\bar{x}$ = 2.520 ± 0.063). The second group included *E. helmutiana*, *E. catorce* (Accession number 5469), *E. olivacea,* and *E. juarezensis* ($\bar{x}$ = 2.216 ± 0.047). The third group included *E. caamanoi*, *E. uhlii*, *E. magnifica*, *E. guerrerensis* (Accession number 7526), *E. carnicolor*, *E. zorzaniana*, *E. perez-calixi,* and *E. novogaliciana* ($\bar{x}$ = 1.841 ± 0.163). The fourth group included *E. pallida*, *E. gibbiflora*, *E. guerrenesis* (Accession number 7521), *E. multicaulis*, *E. triquiana*, *E. altamirae*, *E. dactylifera,* and *E. longiflora* ($\bar{x}$ = 1.221 ± 0.101). The last group included *E. roseiflora* and *E. cupreata* ($\bar{x}$ = 0.752 ± 0.088). Species from the Angulatae series presents, on average, the largest cycle value ($\bar{x}$ = 2.451 ± 0.183), followed by the Racemosae series ($\bar{x}$ = 2.155 ± 0.141) and species of the Gibbiflora series show the lowest values on average ($\bar{x}$ = 1.412 ± 0.428). 

### 3.4. Correlation Polyploidy, Chromosome Number, and 2C DNA Content

**Figure 7:**
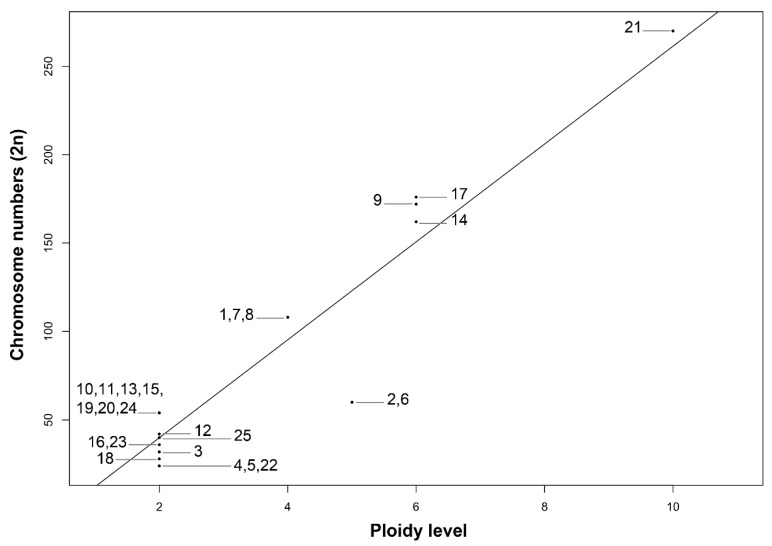
Correlation between 2*n* and the ploidy level in 23 species of *Echeveria*. (1) *E. altamirae*, 2*n* = 108 = 4*x*. (2) *E. caamanoi*, 2*n* = 60 = 5*x*. (3) *E. carnicolor*, 2*n* = 36 = 2*x*. (4) *E. catorce*, 2*n* = 24 = 2*x*. (5) *E. catorce*, 2*n* = 24 = 2*x*. (6) *E. cuicatecana*, 2*n* = 60 = 5*x*. (7) *E. cupreata*, 2*n* = 108 = 4*x*. (8) *E. dactylifera*, 2*n* = 108 = 4*x*. (9) *E. gibbiflora*, 2*n* = 172 = 6*x*+10. (10) *E. guerrerensis*, 2*n* = 54 = 2*x*. (11) *E. guerrerensis*, 2*n* = 54 = 2*x*. (12) *E. helmutiana*, 2*n* = 42 = 2*x*. (13) *E. juarezensis*, 2*n* = 54 = 2*x*. (14) *E. longiflora*, 2*n* = 162 = 6*x*. (15) *E. magnifica*, 2*n* = 54 = 2*x*. (16) *E. multicaulis*, 2*n* = 32 = 2*x*; (17) *E. novogaliciana*, 2*n* = 176 = 6*x*+14; (18) *E. olivacea*, 2*n* = 28 = 2*x*. (19) *E. pallida*, 2*n* = 54 = 2*x*. (20) E*. perezcalixii*, 2*n* = 54 = 2*x*. (21) *E. roseiflora*, 2*n* = 270 = 10*x*. (22) *E. schaffneri*, 2*n* = 24 = 2*x*. (23) *E. triquiana*, 2*n* = 32 = 2*x*. (24) *E. uhlii*, 2*n* = 54 = 2*x*. (25) *E. zorzaniana*, 2*n* = 40 = 2*x*. Correlation results were statistically significant (*p* < 0.001). Numbers in the graphic correspond to species numbers in Table 2 and Figure 1, Figure 2, Figure 3 and Figure 4.

**Figure 8:**
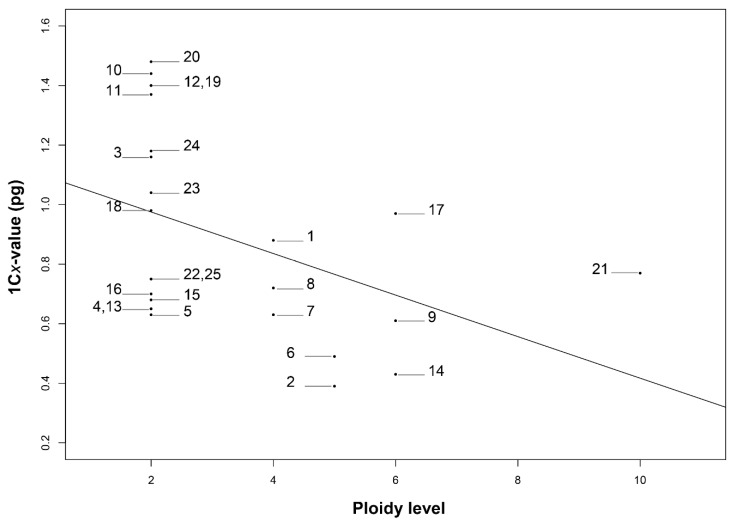
Correlation between the DNA 1C*x*-value and ploidy level in 23 species of *Echeveria*. (1) *E. altamirae,* 1C*x* = 0.88 pg. (2) *E. caamanoi,* 1C*x* = 0.39 pg. (3) *E. carnicolor,* 1C*x* = 1.16 pg. (4) *E. catorce,* 1C*x* = 0.65 pg. (5) *E. catorce,* 1C*x* = 0.63 pg. (6) *E. cuicatecana,* 1C*x* = 0.49 pg. (7) *E. cupreata,* 1C*x* = 0.63 pg. (8) *E. dactylifera*, 1C*x* = 0.72 pg. (9) *E. gibbiflora,* 1C*x* = 0.61 pg. (10) *E. guerrerensis,* 1C*x* = 1.44 pg. (11) *E. guerrerensis*, 1C*x* = 1.37 pg. (12) *E. helmutiana,* 1C*x* = 1.40 pg. (13) *E. juarezensis,* 1C*x* = 0.65 pg. (14) *E. longiflora,* 1C*x* = 0.43 pg. (15) *E. magnifica,* 1C*x* = 0.68 pg. (16) *E. multicaulis,* 1C*x* = 0.70 pg. (17) *E. novogaliciana,* 1C*x* = 0.97 pg. (18) *E. olivacea,* 1C*x* = 0.98 pg. (19) *E. pallida,* 1C*x* = 1.40 pg. (20) *E. perezcalixii,* 1C*x* = 1.48 pg. (21) *E. roseiflora,* 1C*x* = 0.77 pg. (22) *E. schaffneri,* 1C*x* = 0.75 pg. (23) *E. triquiana,* 1C*x* = 1.04 pg. (24) *E. uhlii,* 1C*x* = 1.18 pg. (25) *E. zorzaniana,* 1C*x* = 0.75 pg. Correlation results were statistically significant (*p* < 0.03 with *r* = −0.430).

There was a positive and high correlation between the ploidy level and the chromosome number (2*n*) (*r* = 0.93, *p* < 0.001, Figure 7). On the other hand, a negative correlation between polyploidy and the 1C*x*-value (*r* = −0.43, *p* = 0.03. Figure 8) was observed, which implies that as polyploidy level increases, the chromosome number also increases, but the DNA content of one monoploid genome decreased, suggesting a reduction of the DNA content of chromosomes in plants with the highest ploidy level.

## 4. Discussion

### 4.1. Endemism, Chromosome Numbers, and Polyploidy in Echeveria

The species of the genus *Echeveria* included in this study come from 9 States of Mexico; 16 of the 23 species were endemic which corresponds to 69.6% (Table 1 and Table 2). Of the total endemic species, 11 were collected in the Oaxaca State, 2 in Guerrero, 1 in Puebla, 1 in Michoacán and 1 more in Veracruz. The high number of endemic species in the state of Oaxaca is consistent with the observations of other authors, who consider Oaxaca as the state with the highest diversity and endemism of this genus in Mexico [7]. In fact, Mexico presents a high percentage of endemism for this genus because, of the 170 species described, approximately 140 are endemic, which corresponds to 85% of endemism distributed in different localities in Mexico [3,8].

The 23 studied species of *Echeveria* belonged to 7 of the 17 series of the genus (Table 1). A great diversity of chromosome numbers (2*n*) was observed within each one of the series and also when those values were compared between the series (Table 1 and Table 2). The chromosome number (2*n*) of 16 *Echeveria* species are reported for the first time, which corresponds to 69.6% of the total species studied in the present investigation (Table 2, Figure 1, Figure 2, Figure 3 and Figure 4). 

From the Gibbiflorae series, 13 species were analyzed: 7 from the state of Oaxaca, 2 from Guerrero, 1 from Zacatecas, 1 from Jalisco, 1 from Michoacán, and 1 from Durango. This series presents high diversity in terms of the chromosome numbers; 6 of the 13 species studied in this series are diploids, five of which (*E. perezcalixii*, *E. guerrerensis*, *E. pallida*, *E. magnifica,* and *E. juarezensis*) had 2*n* = 2*x* = 54 with *x* = 27 while *E. triquiana* had 2*n* = 2*x* = 32 with *x* = 16. Regarding this species, Uhl [13] reported three diploid populations of *E. juarezensis* with *n* = 27, similar to the one studied here; the first one was found in Sierra de Juarez, the second one came from Sola de Vega locality, and the third population belonged to Zoquiapan; all these localities are in Oaxaca, Mexico. Uhl [13] informed gametic numbers about a tetraploid population of *E. juarezensis* with *n* = 54, from San Felipe, Oaxaca. Similarly, Uhl [13] analyzed other triploid populations of *E. juarezensis* from Sierra de Juarez, Oaxaca, and another hybrid of *E. juarezensis*, with irregular meiosis from Sierra de Juárez, Oaxaca. Uhl [13] also mentioned one population of *E. pallida* collected in Oaxaca, also a diploid with *n* = 27 and two other tetraploid-cultivated populations with *n* = 54 of *E. pallida*, one from Oaxaca, and another from Mexico City.

The three tetraploid species analyzed in this research (*E. altamirae*, *E. dactyliffera,* and *E. cupreata*) belong to the Gibbiflorae series, and the three of them have 2*n* = 4*x* = 108 and *x* = 27. Uhl [13] pointed out that one population, also a tetraploid of *E. dactylifera,* from Durango state in Mexico, *n* = 54, like the one reported in this investigation. On the other hand, from this same series, we analyzed a hexaploid species (*E. longiflora*) with 2*n* = 6*x* = 162 and *x* = 27; this species is like a hexaploid population of *Echeveria scopolorum* (2*n* = 6*x* = 162) reported by Uhl [13] which was collected on the road from Michoacán to Veracruz, Mexico. The highest chromosome number of all the species analyzed in this study was found in *E. roseiflora*, which had 2*n* = 10*x* = 270 with *x* = 27 and belongs to the series Gibbiflorae.

Within the Gibbiflorae series, we found two polyploid–aneuploid hybrids: *E. gibbiflora* with 2*n* = 6*x* +10 = 172, (*x* = 27) and *E. novogaliciana* with 2*n* = 6*x* + 14 = 176 (*x* = 27). *E. gibbiflora* was collected in Tlaxiaco, Oaxaca, Mexico while *E. novogaliciana* came from Jalisco, Mexico (Table 1 and Table 2). Uhl [13] mentioned diploid populations of *E. gibbiflora n* = 27, from Puente Río Turundeo, Michoacan, and the other one with *n* = 27 + 1 (with a trisomic chromosome), was collected 5 Km from Tuxpan, Veracruz. Uhl [13] also found 5 tetraploid populations *n* = 54 of *E. gibbiflora* collected in the following localities: (1) Amanalco, Mexico state; (2) Cerro Teresona, in Toluca, Mexico state; (3) Tenancingo, Mexico state; (4) Ahuatenco, Morelos state and (5) Road to Cuernavaca, Morelos state. Uhl [13] reported one tetraploid population *n* = 54 + 7B of *E. gibbiflora*, from Tenancingo in Mexico state. Finally, Uhl [15] reported *Echeveria chiclensis* (*n* = 50–55), and *E. gigantea*, also with (*n* = 54) [19]. Polyploid–aneuploid species of *Echeveria fulgens* (*n* = 135) from Valle de Bravo and Temascaltepec, Mexico state, like *E. gibbiflora* and *E. novogaliciana,* have been reported by Uhl [13]. In addition, this author pointed out two aneuploidy species, one *n* = ca. 162 and the other *n* = ca. 135, from Angangueo and San Lorenzo, in Michoacan state, and near Paricutin volcano, respectively.

From the Racemosae series, four species were analyzed in the present investigation; three of them were from Oaxaca and one was from Veracruz, and all of them were endemic and a diploid. However, all had different chromosome numbers; thus, *E. olivacea* presented 2*n* = 2*x* = 28 and, *E. carnicolor* had 2*n* = 2*x* = 36, *E. helmutiana* had 2*n* = 2*x* = 42, and *E. uhlii* had 2*n* = 2*x* = 54. (Table 1 and Table 2, Figure 1c, Figure 2f, Figure 3f and Figure 4f). The chromosome number we found for *E. carnicolor* corresponds to what Uhl [14] reported for 2 populations of this species, including *n* = *x* = 18 and another *n* = 18 + 2 or 3 extra chromosomes, also from Veracruz, Mexico. Uhl [14,17] reported *Echeveria diffractens n* = 18 and *Echeveria racemosa n* = 18; these species presented 2*n* = 2*x* = 36, *x* = 18 is the same as *E. carnicolor*. On the other hand, regarding *E. helmutiana*, this was the first count for the species with 2*n* = 2*x* = 42 (Table 1, Figure 2f); Uhl did not report *n* or 2*n* in *E. helmutiana*. However, he reported *n* = 42, *x* = 21 for *Echeveria multicolor* from Merida Venezuela, South America to be similar *to E. helmutiana* [12]. Uhl [11,13] reported two populations of *Echeveria carmenae*; one of these populations was a diploid *n* = 21 like *E. helmutiana*, and the other population was a triploid 3*x* + 2 = 65, both from Oaxaca, Mexico similar to *E. uhlii* in this study. Uhl [11,14,16,17] reported diploid populations with *n* = 27 in: *Echeveria acutifolia*, *E. colorata*, *E. derenbergii*, *E. grisea*, *E. lilacina*, *E. lindsayana*, *E. nayaritensis*, and *E. purpurosum*. 

From the Angulatae series, two species were analyzed, *E: shaffneri* and *E. catorce* (two localities were studied of the latter). The three populations were collected in San Luis Potosí and were diploids with 2*n* = 2*x* = 24 with *x* = 12. These numbers were coincident with results reported by Uhl for these species from the same locality of the state of San Luis Potosi, Mexico [13]. Regarding the Echeveria series, only *E. zorzaniana* was analyzed, which was collected in Oaxaca and was a diploid; this was the only species with 2*n* = 2*x* = 40 and *x* = 20 in the present study and corresponds to the first count informed for this species (Table 2, Figure 4g). Nevertheless, Uhl [19] reported *n* = 20 for *Echeveria secunda*, from the central area of Mexico. 

From the Nudae series, *E. multicaulis* from Guerrero was the only species analyzed, being a diploid with 2*n* = 2*x* = 32. For this same species, Uhl [12,16,17] found one diploid population, and one triploid population (2*n* = 3*x* + 6 = 48) in Merida, Venezuela (South America). Both *E. caamanoi* from the Urbinae series (collected in Puebla), and *E. cuicatecana* from the Pruinosae series (collected in Oaxaca), turned out to be 5*x* with 2*n* = 5*x* = 60. It is important to mention that in this study that we confirmed the 2*n* = 60 (5*x*) for *E. cuicatecana*, which was previously reported by Reyes et al. [63]. The basic chromosome number *x* = 12 is frequent within the genus *Echeveria*. In fact, Uhl has also reported this number for other species of *Echeveria*, such as *E. lutea*, *E. secunda*, *E. strictiflora,* and *E. tenuifolia*, from some localities of San Luis Potosi [11,13,14,16,17]. 

In this investigation, we found 13 different chromosome numbers: 2*n* = 24, 28, 32, 36, 40, 42, 54, 60, 108, 162, 172, 176, and 270, and 2 species that show different levels of polyploidy–aneuploidy. In fact, evidence from the literature indicates a wide diversity of chromosome numbers within the genus *Echeveria.* Nevertheless, some chromosome numbers as those of *E. carmenae* [11,13] with chromosome number 2*n* = 65 has not been reported in this study; in addition to this, there are some polyploids–aneuploids showing chromosome numbers different to those reported here, for example, *n* = 320 in *E. bakery*, *n* = 119 + 2 in *E. chiclensis* from Ecuador to Argentina [15], which suggests that the genus *Echeveria* has experienced a strong chromosomic evolution. With respect to the ploidy level, it is well known that the whole genome duplication (WGD) has led to an increase in species richness [30]. Polyploidy as a mechanism can generate individuals capable for the adaptation and colonization of new ecological niches, favoring the survival and reproduction of individuals more capable to adapt to new environments, with respect to diploid individuals [47,65]. In this investigation, a positive and strong correlation between the polyploidy and the chromosome number (*r* = 0.93, *p* < 0.0001) in the 25 populations of the 23 species of *Echeveria* was analyzed (Figure 5). Although this correlation could be expected, its verification in this genus is interesting, especially when Uhl has pointed out the importance that this phenomenon represents in the evolution and adaptation of this genus [5].

Although polyploidization is an important process in the evolution of angiosperms, it has as a consequence during meiosis: there is a high frequency of non-disjunction of sister chromatids, which is due to the fact that sister chromatids associate in multivalents, rather than in bivalents during meiotic prophase I, resulting in the formation of aneuploid gametes [66]. On the other hand, the cellular machinery and the entire organism become unviable with indefinite increases in DNA and chromosomes, so one of the processes that polyploidization causes is so-called diploidization, which occurs thanks to a series of massive chromosomal rearrangements, including reductions in the number of chromosomes and a significant loss of repetitive sequences and duplicated genes [65,67]. Thanks to these processes, there is a reduction in the size of the genome, which generates enormous variation (for example in Asteraceae) [65,68]. Therefore, the diploidization phenomenon involves mechanically diverse processes, which operate together and in the long term result in the generation of descendants that behave like normal diploids during meiosis, but that reflect vestiges of past polyploidy events in their genomes [67]. Diploidization has been evident in autopolyploid plants, as was demonstrated in *Zea mays* [69] and has been observed in autotetraploid plants of *Zea perennis* [70], *Festuca* sp. [71], and autotetraploid cytotypes of *Gibasis schiedeana* [72].

In the present investigation, three tetraploid species were found with *n* = 54 (*E. altamirae*, *E cupreata* and *E. dactilifera*), two pentaploids with *n* = 30 (*E. caamanoi* and *E. cuicatecana*) and a 6*x* species with *n* = 81 (*E longiflora*), in addition to two polyploid aneuploids, one 6*x* + 10 with *n* = 86 (*E. gibbiflora*) and another 6*x* + 14 (*E. novogaliciana*), while in diploid species values of *n* = 12, 14, 16, 18, 20, 21 and 27 were observed. On a larger scale, Uhl [14,16,17] proportionated values of *n* = 12–100, 119, 135–162 and polyploid up to 13*x*-, 20*x,* and 42*x*, mainly in species of *Echeveria* from South America, where he confirmed that polyploidy was the base for the evolution of the chromosome number in the species of *Echeveria*, through diploidization of their genomes. Moreover, Uhl [15] argues that genetic recombination processes in the genomes of polyploid–aneuploid species found in localities across Ecuador and Argentina are due to adaptation to different new environments, when compared to *E. gibbiflora* and *E. novogaliciana* from México. Diploidization processes probably allowed these species to survive in other localities distinct from their original environments Uhl [15].

Because the species belonging to the Gibbiflorae series are the most represented in this study, it is important to point out that high diversity was found in the levels of ploidy and in the number of chromosomes and DNA content, and in fact, the two species that had the highest number of chromosomes and DNA content in this study belong to this series (Table 2). These results coincide with the fact that this series has been reported as highly diversified and widely distributed [7]. The increase in the chromosome number has an advantage in colonizing new environments, as the distribution of this series confirms in Mexico, where it could be hypothesized that the diploidization process was the main mechanism of series diversification. It is relevant to mention that data on the chromosome number and DNA content can help in the phylogenetic resolution of this genus, because even though important molecular studies in the Gibbiflorae series show a monophyletic origin, there are still some phylogenetic relationships that need to be resolved [73]. 

### 4.2. Nuclear DNA Content and Ploidy Levels in Echeveria

It is important to mention that there are no reports in the literature for the genome size (nuclear DNA content) for any species of *Echeveria*. Therefore, results of this study are the first reported for the studied species of this genus.

We obtained the DNA content of parenchyma tissue from 25 populations of 23 *Echeveria* species (Table 2); the highest value of 2C that we found was in *E. roseiflora* with 7.70 pg, and 1C*x* of 0.77pg, while the lowest value was found in 1 of the 2 populations of *E. catoce* with 2C = 1.26 pg, and 1C*x* = 0.63 pg. Although there are no data in the literature on the size of the genome in the genus *Echeveria*, there are some records of Mexican species of other genera of the Crassulaceae family; one of them is that of *Sedum suaveolens* from Durango with 2C = 18.20 pg [40] which, as mentioned before, is the highest chromosome number recorded in angiosperms. On the other hand, *Sedum burrito*, which is cultivated in Guadalajara and Veracruz, Mexico, but is not known in the wild, was 2C = 1.3 pg [40,74]. Other species of the genus *Graptopetalum,* which is phylogenetically closely related to the genus *Echeveria* [7], are *G. macdougallii* from Oaxaca with 2C = 6.70 pg [40] and *G. bellum* from Chihuahua with 2C = 8.40 pg [40,75]. As can be seen, there is a wide variation in the size of the genome in these genera and in both, some 2C values are even higher than those found in this investigation.

It is also relevant to compare the size of the genome with the level of ploidy and the number of chromosomes, but of the Mexican genera mentioned, these data are only available for *S. suaveolens*, which is 20*x* with 2*n* = 640 and 2C = 18.20 pg [12,40,75]. Meanwhile, *E. roseiflora,* which was the species with the highest level of ploidy in this study, was 10*x* with 2*n* = 270 and 2C = 7.70 pg. However, with respect to European species, *Sedum forsteriaum* was reported as a diploid species with 2*n* = 24 and 2C = 0.92 pg [40,76] while we observed 2C = 1.26 pg and 1.29 pg for *E catorce* and 2C = 1.50 pg for *E. shaffneri,* both diploid species with 2*n* = 24. Another European species, *S. sediforme*, *is* also 2*x* but with 2*n* = 32 and 2C = 1.16 pg [40,76], it can be compared with *E. triquiana* and *E. multicaulis,* both diploids with 2*n* = 32 but with 2C = 2.07 pg and 2C = 1.40 pg, respectively. 

In general, within the species analyzed by us, the 2C values in diploid species varied between 2.96 pg in *E. perezcalixii* and 1.26 pg in *E.catorce*, while in the tetraploid species, values between 2C = 3.54 pg in *E. altamirae* and 2C = 2.50 pg in *E. cupreata* were observed. In *E. cuicatecana* and *E. caamanoi*, both pentaploids, values 2C = 2.44 pg and 2C = 1.96 pg were observed, respectively, and in *E. longiflora,* which is hexaploidy, it was 2C = 2.54 pg. Of the two polyploid–aneuploid species, *E. gibbiflora* (6*x* + 10) presents 2C = 3.68 pg and *E. novogaliciana* presents (6*x* + 14) 2C = 5.81 pg. These data reflect a certain tendency to increase in the 2C value as the ploidy level increases, but it is highly variable because finally, it also depends on the basic chromosome number and chromosome size for each species.

Although a positive significant correlation was observed between ploidy level and chromosome number, a negative significant correlation between ploidy level and the 1C*x*-value was observed. The decrease in the monoploid genome size as polyploidy level increases is a process known as genome downsizing [77] and can be explained as a strategy for the reduction in the number of chromosomes and a significant loss of both, repetitive sequences and duplicated genes related to the diploidization process [65,67]. Wang et al. [77] propose that genome downsizing may be a byproduct of various processes that give rise to smaller genomes, which could offer a selective advantage as an emergent property. As Wendel [67] has mention, angiosperms history includes many multiple events of polyploidy and reduction in chromosome numbers through massive rearrangement, which cause the reduction in genome size [65,78].

### 4.3. Endopolyploidy 

As there are not previous reports in the literature of the endopolyploidy in any species of *Echeveria*, results on endopolyploidy in this investigation are the first records for the studied species of this genus. However, Zonneveld [79] mentions the presence of an endopolyploidy in some genera belonging to the Crassulaceae family, such as: *Sedum*, *Crassula*, *Sempervivum,* and *Graptopetalum*, which presented endoploidy levels between 8C and 32C.

In this investigation, we observed the existence of endopolyploidy patterns in all the analyzed species, but with differences in the number of endocycles and percentage of nuclei in each endocycle, and observed species that presented from one to four endocycles, which represents endopolyploidy levels from 8C to 64 C (Table 3) in the different species. Also, the presence of two levels of endopolyploidy was observed within the same species and population in *E. roseiflora*, *E. longiflora*, *E.olivacea*, *E. juarezensis*, *E. perezcalixii*, and *E catorce* (Accession number 5469) (Table 4). Variations in endopolyploidy levels and in the number of nuclei in each endocycle were reflected in the cycle value that showed a range between 0.682 and 2.562 (Table 3 and Table 4; Figure 1d, Figure 3a,b,f and Figure 4b,c).

Endopolyploidy represents a metabolic adaptation of plants that favors the survival and reproduction of individuals living in arid environments [80,81] and it is considered an emergent response in species that live in these environments, as it has been observed in species of *Mammillaria* [34] and *Opuntia* [24]. Moreover, it has been suggested that the biological significance of endopolyploidy is to provide a high DNA content to sustain the demand of DNA synthesis in the cells of species with small genomes and with specialized functions [82], such as in the endosperm cells of *Arum maculatum* specialized in nutrition, where the presence of an endopolyploidy pattern with levels up to 24,576C (13 endocycles) has been reported [33]. However, at least for certain tissues, this assumption seems unlikely because the proportionality between the ploidy level and genome size has been inconsistent in some plant families and they do not have a relationship that indicates the need for a particular amount of DNA to maintain cell function [33,60].

The genus *Echeveria* has a wide distribution and presents different mechanisms that allow them to adapt to different environments. Endopolyploidy has been suggested to play an important role in how plants cope with situations that constitute some form of stress for them. Recently, information has been generated that shows that stress itself, whether biotic or abiotic, can trigger endopolyploidy as part of a plant response to stress, helping to mitigate its effect on the plant. For example, it allows continued growth by endopolyploidy induced cell expansion when growth via cell division is inhibited by a low temperature, or by inducing the formation of larger cells via endopolyploidy which can reduce the number of guard cells and consequently the loss of water, or providing a layer of cells that absorb potentially damaging light and thus protecting the plant against possible damage caused by light [50,53]. Endopolyploidy could also be related to the production of mucus (polysaccharides) to retain moisture, as Zonnevelt [79] suggests. Furthermore, endopolyploidy level is variable to some degree and may even present differences between individuals or populations of the same species in response to different environmental conditions [50,61], as seen in six of the species analyzed in this investigation (*E. catorce*, *E. olivacea*, *E. juarezensis*, *E. perez-calixi*, *E. longiflora,* and *E. roseiflora*). In fact, the cycle values obtained reflect this variability within the same species and population, and can be deduced from the results of the Tukey–Kramer test, where it was observed that several species were grouped within the same block, despite the fact that the range of variation of the cycle values was wide.

## 5. Conclusions

A high variability of the number of chromosomes (2*n* and *x*) could be observed among the species of the genus *Echeveria* analyzed; 13 different chromosome numbers were found, and 2*n* = 54 and *x* = 27 were the most frequent. This variability was particularly observed in the diploid species that presented 7 different basic chromosome numbers that were *x* = 12, 14, 16, 18, 20, 21 and 27, the last one being the most frequent. Meanwhile, in the pentaploid species *x* = 12, and in tetraploid, hexaploid, decaploid, and hexaploidy–aneuploid species *x* = 27 was observed. The chromosome number (2*n*) of 16 species of the genus is reported for the first time in this research, which coincided with some of those reported by Uhl for other species.

Results of genome size were reported for the first time in 25 populations of 23 species of *Echeveria* in this research. The lowest value was observed in *E. catorce* where 2*n* = 2*x* = 24 with 2C DNA = 1.26 pg; the highest value of 2C DNA = 7.70 pg was observed in *E. roseiflora* 2*n* = 10*x* = 270. Moreover, the presence of endopolyploidy pattern in the leaf parenchyma of species of *Echeveria* was also observed for the first time in this genus, corresponding to cells with endopolyploidy levels from 8C up to 64C and cycle values between 0.690 and 2.562 in the different species of *Echeveria* analyzed.

The variability observed in the basic number of chromosomes, as well as the presence of different ploidy levels and two polyploid–aneuploid species in the analyzed species, coincide with several of Uhl’s [13,14,15] observations in various species of this genus from Mexico and from Central and South America. These data, together with the positive correlation found between ploidy level and the number of chromosomes, and the negative correlation between polyploidy levels and 1C*x*- value, could provide evidence of the existence of different polyploidization–diploidization cycles in each species, and as was suggested by Wendel [67], generate changes in the phenotypes on which natural selection acts, resulting in the diversification of the species. Additionally, we found that the species analyzed in this research were polysomatic, and it has been suggested that endopolyploidy allows plants to adapt to various types of stresses and survive in different environments [50,60,61]. Thus, all these processes acting simultaneously may be the cause of the high degree of endemism as well as the wide diversification and distribution of this genus. Although no clear differences could be found between the different series of the genus included in this study, the obtained results are relevant in studies about systematics, phylogeny, karyotype evolution [67,83], and improvement programs of the genus *Echeveria* and ornamental hybrid plant production [84]. 

## Figures and Tables

**Table 1:** Provenance of *Echeveria* species and populations examined in this study.

No.	Taxon	Serie	Accession Number	Locality
1	*E. altamirae*	Gibbiflorae	JE-7548	San Vicente Muñú, Oaxaca, Mexico. Near Anama. 17°22′10″ N 97°26′42″ W. 1828 m asl
2	*E. caamanoi*	Urbinae	JE-8311	Ixtacamaxtitlan, Puebla, Mexico. Course path from Ixtacamaxtitlan–Texocoixpan. 19°36′52.5″ N 97°48′47.7″ W. 2347 m asl
3	*E. carnicolor*	Racemosae	JE-8374	Tenampa, Veracruz, Mexico. Barranca de tenampa. 19°15′27.7″ N 96°52′59″ W. 762 m asl
4	*E. catorce*	Angulatae	JE-5469	Catorce, San Luis Potosí, Mexico. 0.5 Km Dirt Road Catorce-Vanegas. 23°41′40.67″ N 100°53′18.88″ W. 2690 m asl
5	*E. catorce*	Angulatae	EK-3223	Catorce, San Luis Potosí, Mexico. Matehuala-Real de Catorce. 23°41′30.9″ N 100°53′15.2″ W. 2713 m asl
6	*E. cuicatecana*	Pruinosae	JP-584	San Juan Bautista Cuicatlán, Oaxaca, Mexico. Between Santo Dominguito and San Juan Tonaltepec. 17°41′21.5″ N 96°53′28.9″ W. 800 m asl
7	*E. cupreata*	Gibbiflorae	JE-6807	San Vicente Lachixio, Oaxaca, Mexico. Km. 20 between the caves of San Sebastian and Vicente Guerrero. 16°41′6.2″ N 96°56.5″ W. 2093 m asl
8	*E. dactylifera*	Gibbiflorae	EK-2603	San Dimas, Durango, Mexico. Mexico to Durango Road, Km. 172.5. 23°39′ N 105°47′9.9 W. 2493 m asl
9	*E. gibbiflora*	Gibbiflorae	EK-3427	Tlaxiaco City, Oaxaca, Mexico. Putla-Nundaco and Atatlahuca. 17°12′0.7″ N 97°43 39.4″ W. 2125 m asl
10	*E. guerrerensis*	Gibbiflorae	JE-7521	San Miguel Totolapan, Guerrero México. 3 Km NW from Station Toro Muerto. 17°34′42 N 100°17′0″ W. 2731 m asl
11	*E. guerrerensis*	Gibbiflorae	JE-7526	Zihuatanejo de Azueta, Guerrero, Mexico Zihuatanejo to Ciudad Altamirano road. 17°56′2″ N 101°17′1″ W. 1446 m asl
12	*E. helmutiana*	Racemosae	JE-7075	Santiago Juxtlahuaca, Oaxaca, Mexico. Dirt Road Juxtlahuaca-Yucunicoco. 17°17′44″ N 97°59′9.8″ W. 2476 m asl
13	*E. juarezensis*	Gibbiflorae	JE-7538	Santa Catarina Ixtepeji, Oaxaca, Mexico. 10.5 Km South from “la Cumbre”. Km. 191.5 of the Oaxaca-Tuxtepec Road. 17°10′157″ N 96°36′15″ W. 2726 m asl
14	*E. longiflora*	Gibbiflorae	JE-6770	Taxco, Guerrero México. Km. 21, of the Taxco-Ixcateopan Road. 18°31′54.5 N 99°42′54.9″ W. 2293 m asl
	*E. longiflora*	Gibbiflorae	JE-6923	Ixcateopan de Cuahtemoc, Guerrero, Mexico. Near “Los Naranjos” 3 Km. far from Ixcateopan. 18°31′32.4″ N 99°44′42″ W. 2256 m asl
	*E. longiflora*	Gibbiflorae	JE-6013	Taxco, Guerrero, Mexico. “Cruz Verde, El Puerto” between Taxco and Ixcateopan, 17 Km. far from Taxco.
15	*E. magnifica*	Gibbiflorae	JE-6270	San Juan Ozolotepec, Oaxaca, Mexico. “Rio Grande” 2 Km North-Northeast from San Juan Ozolotepec. 16°7′37″ N 96°15′18″ W. 1942 m asl
16	*E. multicaulis*	Nudae	JE-7501	Leonardo Bravo, Guerrero, Mexico. “Filo de Caballo” in the way to “Cruz de Ocote”. 17°37′0″ N 99°50′30″ W. 2455 m asl
17	*E. novogaliciana*	Gibbiflorae	JE-6580	Calvillo Municipality, Aguascalientes, Mexico. “Barranca el Montoro” between “Potrero de Lopez” and Milpillas. 21°59′48.87″ N 35′30.8″ W. 2343 m asl
	*E. novogaliciana*	Gibbiflorae	JE-6823	Zapopan, Jalisco, Mexico. Collin Hill, SE from football “las Chivas” stadium. 20°39′45.7″ N 103°27′39.3″ W. 1872 m asl
18	*E. olivacea*	Racemosae	JE-6402	San Miguel Tenango Municipality, Oaxaca, Mexico. San Pedro Hill. 16°16′38″ N 95°31′44″ W. 1282 m asl
	*E. olivacea*	Racemosae	EK-3899	San Miguel Tenango Municipality, Oaxaca, Mexico. Tehuantepec-Jalapa de Marquez to San Miguel Tenango. 16°16′41.3″ N 95°31′44.5″ W. 1302 m asl
19	*E. pallida*	Gibbiflorae	JE-6475	San Juan Guichicovi, Oaxaca, Mexico. 4 Km West from “Hierba Santa”. Near to the train track and Malatenco riverbank. 16°17′30″ N 95°1′30″ W. 100 m asl
20	*E. perezcalixii*	Gibbiflorae	PCR-6322	Teul de Gonzalez Ortega, Zacatecas, Mexico. Conejo-Milpillas Road. 21°21′4″ N 103°33′59″ W. 1700 m asl
21	*E. roseiflora*	Gibbiflorae	JE-6744	Morelia, Michoacan, Mexico. “Cañada del Cerro Azul”, 3 Km South from “San Miguel del Monte”. 19°34′57.6″ N 101°7′38.7″ W. 2253 m asl
	*E. roseiflora*	Gibbiflorae	JE-6821	“La Mascota” Municipality, Jalisco, Mexico. at 1.5 Km al oeste de Juanacatlan. 20°35′42.9″ N 104°42′29″ W. 2235 m asl
22	*E. schaffneri*	Angulatae	OZ-54	Guadalcazar, San Luis Potosi, Mexico. Path to Santa Rita del Rocio to “El Jaujal”, next to the path. 23°3′35″ N 100°17′47″ W. 1641 m asl
23	*E. triquiana*	Gibbiflorae	JE-6396	San Sebastian Tecomaxtlahuaca, Oaxaca, Mexico. “Laguna Encantada”, 5 Km North from Santiago Juxtlahuaca. 17°22′1″ N 98°1′26″ W. 1703 m asl
24	*E. uhlii*	Racemosae	JE-6437	San Pedro Nopala, Oaxaca, Mexico. River Elite, at North from San Pedro Nopala. 17°55′13″ N 97°26′27″ W. 2220 m asl
	*E. uhlii*	Racemosae	JE-8553	San Pedro Nopala, Oaxaca, Mexico. “Cañada del Cerro Pericón”, Nopala. 17°50′7.2″ N 97°33′4.1″ W. 2512 m asl
25	*E. zorzaniana*	Echeveria	JE-7237	Villa Diaz Ordaz, Oaxaca, Mexico. 3 Km. North from San Miguel del Valle, dirty road from “El Carrizal”-Diaz Ordaz. 17°4′39″ N 96°24′9″ W. 2758 m asl

The species *E. catorce* and *E. guerrensis* have two collection numbers, but each one corresponds to a different population, so they were analyzed independently. The species *E. longiflora*, *E. novogaliceana*, *E. olivacea*, *E. roseiflora,* and *E. uhlii* have more than one collection number because they were collected on different dates or by different collectors, but belong to the same population, so they were analyzed together.

**Table 2:** Chromosome number, ploidy level, internal standards used and genome size, and 2C DNA Tukey-Kramer test of species of *Echeveria*.

Taxon	Chromosome Number			Internal Standard	2C DNA Content (pg) ($\bar{x}\;\pm$ SE)	1C*x*-Value Mpb	Tukey’s Grouping								
	2*n*	*x*	Ploidy Level												
*E. roseiflora*^δ^*	270	27	10*x*	*P. sativum*	7.70 ± 0.10	753	a								
*E. novogaliciana* *	176	27	6*x* + 14	*P. sativum*	5.81 ± 0.06	947		b							
*E. gibbiflora*	172	27	6*x* + 10	*Z. mays*	3.68 ± 0.05	599			c						
*E. altamirae*^δ^*	108	27	4*x*	*Z. mays*	3.54 ± 0.10	865			c	d					
*E. perezcalixii* *	54	27	2*x*	*S. lycopersicum*	2.96 ± 0.05	1447				d	e				
*E. dactylifera*	108	27	4*x*	*Z. mays*	2.90 ± 0.10	709				d	e	f			
*E. guerrerensis*^δ^*	54	27	2*x*	*P. sativum*	2.88 ± 0.10	1408				d	e	f			
*E. helmutiana*^δ^*	42	12	2*x*	*P. sativum*	2.81 ± 0.10	1374				d	e	f			
*E. pallida* ^δ^	54	27	2*x*	*S. lycopersicum*	2.79 ± 0.07	1364				d	e	f			
*E. guerrerensis*^δ^*	54	27	2*x*	*P. sativum*	2.73 ± 0.10	1335				d	e	f	g		
*E. longiflora*^δ^*	162	27	6*x*	*P. sativum*	2.54 ± 0.06	414					e	f	g		
*E. cupreata*^δ^*	108	27	4*x*	*P. sativum*	2.50 ± 0.07	611					e	f	g		
*E. cuicatecana*^δ^°	60	12	5*x*	*Z. mays*	2.44 ± 0.05	477						f	g		
*E. uhlii*^δ^*	54	27	2*x*	*Z. mays*	2.36 ± 0.04	1154						f	g	h	
*E. carnicolor* ^δ^	36	18	2*x*	*Z. mays*	2.31 ± 0.10	1130						f	g	h	
*E. triquiana*^δ^*	32	16	2*x*	*Z. mays*	2.07 ± 0.10	1012							g	h	
*E. olivacea*^δ^*	28	14	2*x*	*Z. mays*	1.96 ± 0.02	958								h	
*E. caamanoi*^δ^*	60	12	5*x*	*Z. mays*	1.95 ± 0.06	381								h	
*E. schaffneri* *	24	12	2*x*	*Z. mays*	1.50 ± 0.09	733									i
*E. zorzaniana*^δ^*	40	20	2*x*	*Z. mays*	1.49 ± 0.00	729									i
*E. multicaulis*	32	16	2*x*	*S. lycopersicum*	1.40 ± 0.07	685									i
*E. magnifica*^δ^*	54	27	2*x*	*S. lycopersicum*	1.36 ± 0.05	665									i
*E. juarezensis* ^δ^	54	27	2*x*	*S. lycopersicum*	1.31 ± 0.06	641									i
*E. catorce* *	24	12	2*x*	*S. lycopersicum*	1.29 ± 0.02	631									i
*E. catorce* *	24	12	2*x*	*S. lycopersicum*	1.26 ± 0.02	616									i

^δ^ = Endemic species [3,8], *x* = basic chromosome, 1 pg = 978 Mbp [37], 1C*x*-value represents DNA content of one monoploid genome with chromosome number *x* [39], * = 2*n* first reported for *Echeveria*, ° = 2*n E. cuicatecana* informed as a new species by Reyes et al. [63].

**Table 3:** Results of the post hoc Tuckey–Kramer test comparison between the species of series: Gibbiflorae, Racemosae, and Angulatae.

Series	Mean of Chromosome Number	Standard Error	Tukey–Kramer Test		
Gibbiflorae	104.3	15.6	A		
Racemosae	40	29.2		B	
Angulatae	24	33.7			C

**Table 4:** Analysis of endopolyploidy nuclei, number of endocycles, Cycle value, and Cycle value Tukey–Kramer test in 23 *Echeveria* species.

Taxon	Accession Number	Percentage of Nuclei Populations						Number of Endocycles	Cycle Value	Tukey’s Grouping					
		2C	4C	8C	16C	32C	64C								
*E. schaffneri*	54	8.80	25.71	10.55	18.23	28.84	7.87	4	2.562	a					
*E. catorce*	3223	12.49	19.00	11.25	22.68	27.18	7.39	4	2.552	a					
*E. cuicatecana*	584	20.23	11.89	10.83	22.83	28.38	5.84	4	2.448	a					
*E. olivacea*	6402	22.34	13.91	14.54	40.13	9.08		3 or 4 *	2.207	a	b				
*E. catorce*	5469	17.99	23.72	13.82	29.15	15.31		3 or 4 *	2.240	a	b	c			
*E. juarezensis*	7538	27.15	35.07	19.16	12.37	6.26		3 or 4 *	2.155	a	b	c	d		
*E. helmutiana*	7075	12.34	21.65	13.61	31.97	20.44		3	2.265	a	b	c	d	e	
*E. caamanoi*	8311	22.61	25.59	10.96	12.79	23.68	4.37	4	2.024	a	b	c	d	e	
*E. uhlii*	8553	16.77	13.81	30.48	31.62	7.33		3	1.989	a	b	c	d	e	
*E. magnifica*	6270	19.36	18.96	16.42	35.54	9.71		3	1.973	a	b	c	d	e	
*E. guerrerensis*	7526	19.50	27.19	18.70	13.42	16.90	4.30	4	1.939	a	b	c	d	e	f
*E. carnicolor*	8374	26.29	15.11	22.60	27.03	8.97		3	1.773	a	b	c	d	e	f
*E. zorzaniana*	7237	26.34	16.28	17.49	34.34	5.56		3	1.765	a	b	c	d	e	f
*E. perezcalixii*	6322	25.67	24.13	14.29	16.62	19.28		3 or 4 *	1.688	a	b	c	d	e	f
*E. novogaliciana*	6823	16.31	28.60	36.25	18.85			2	1.576	a	b	c	d	e	f
*E. gibiflora*	3427	35.77	27.16	16.38	14.20	6.48		3	1.285	a	b	c	d	e	f
*E. multicaulis*	7501	26.42	32.98	36.26	4.34			2	1.185	a	b	c	d	e	f
*E. dactylifera*	2603	41.23	22.07	18.03	16.91	1.75		3	1.159	a	b	c	d	e	f
*E. pallida*	6475	38.75	18.93	14.12	17.18	11.02		3	1.428		b	c	d	e	f
*E. guerrerensis*	7521	40.24	21.71	16.74	13.97	7.33		3	1.264		b	c	d	e	f
*E. triquiana*	6396	29.81	30.11	31.93	8.15			2	1.184		b	c	d	e	f
*E. altamirae*	7548	39.49	22.33	20.97	17.21			2	1.159			c	d	e	f
*E. longiflora*	6923	42.54	19.67	21.98	15.80			2 or 3 *	1.105				d	e	f
*E. cupreata*	6807	65.65	16.91	6.08	5.55	5.81		3	0.690					e	f
*E. roseiflora*	6744	37.57	48.36	14.07				1 or 2 *	0.815						f

The cycle value was compared with a one-way ANOVA *p* < 0.0001 and Tukey–Kramer test. Same letters indicate no statistical differences using alfa = 0.05. * Species that present two levels of endopolyploidy among individuals of the same population.

**Table 5:** Distribution of the percentage of endopolyploidy nuclei, number of endocycles, and cycle value in 6 *Echeveria* species that present 2 endopolyploidy levels among individuals of the same population.

Taxon	Accession Number	Percentage of Nuclei Populations						Number of Endocycles	Cycle Value
		2C	4C	8C	16C	32C	64C		
*E. catorce*	5469	17.99	23.72	13.82	29.15	15.31		3	2.001
*E. catorce*	5469	13.22	21.31	9.25	21.00	30.99	4.23	4	2.479
*E. olivacea*	6402	22.34	13.91	14.54	40.13	9.08		3	1.997
*E. olivacea*	6402	17.54	19.40	10.71	14.71	31.55	6.10	4	2.416
*E. juarezensis*	7538	27.15	35.07	19.16	12.37	6.26		3	1.355
*E. juarezensis*	7538	15.66	16.68	12.86	16.48	27.96	10.36	4	2.555
*E. perezcalixii*	6322	25.67	24.13	14.29	16.62	19.28		3	1.797
*E. perezcalixii*	6322	40.96	19.81	11.73	11.15	11.35	5.00	4	1.471
*E. longiflora*	6923	42.54	19.67	21.98	15.80			2	1.110
*E. longiflora*	6013	50.84	15.29	9.49	21.91	2.46		3	1.099
*E. roseiflora*	6744	37.57	48.36	14.07				1	0.765
*E. roseiflora*	6744	37.50	33.51	23.96	5.03			2	0.965

## Data Availability

Not applicable.
